# Supplementary material for: Fish Consumption at One Year of Age Reduces the Risk of Eczema, Asthma and Wheeze at Six Years of Age
Source: Nutrients. 2019 Aug 21;11(9):1969. doi: 10.3390/nu11091969 (PMC6770937; doi:10.3390/nu11091969)
Supplement: Supplementary file 1 [file nutrients-11-01969-s001.pdf]

Supplementary Materials

Fish consumption at 1 year of age reduces the risk of eczema, asthma and wheeze at 6 years of age

Torbjørn Øien, Astrid Schjelvaag, Ola Storrø, Roar Johnsen and Melanie Rae Simpson

Table of Contents

§2.1. Study Population: Further details of the PACT study recruitment 2

§3.1. Baseline characteristics of complete cases and drop-outs for each timepoint (Table S1-4) 3

Table S1: Baseline characteristics for participants included in pregnancy analyses and drop-outs 3
Table S2: Baseline characteristics for participants included in breastfeeding analyses and drop-outs 4
Table S3: Baseline characteristics for participants included in 1-year analyses and drop-outs 5
Table S4: Baseline characteristics for participants included in 2-year analyses and drop-outs 6

§3.2. Maternal and Infant Dietary Fish and Cod Liver Oil Intake: Description and Correlations 7

Figure S1: Distribution of fish and cod liver oil consumption 7
Table S5: Correlation / agreement between diet at different time points 8
Table S6: The proportion of families consuming fish at differing combinations of pregnancy, breastfeeding and 1 year 9

§3.3. Maternal fish and cod liver oil intake whilst pregnant allergy related outcomes at 6 years (Table S7-9) 10

Table S7: Association between maternal dietary fish and cod liver oil in pregnancy and allergic disease / symptoms at 6 years: crude and adjusted analyses 10
Table S8: Association between maternal dietary fish and cod liver oil in pregnancy and allergic disease / symptoms at 6 years: stratified by sex 11
Table S9: Association between maternal dietary fish and cod liver oil in pregnancy and allergic disease / symptoms at 6 years: subgroup with data collected during pregnancy 12

§3.4. Maternal fish and cod liver oil intake whilst breastfeeding and allergy related outcomes at 6 years (Tables 10-13) 13

Table S10: Association between maternal dietary fish and cod liver oil whilst breastfeeding and allergic disease / symptoms at 6 years: crude and adjusted analyses 13
Table S11: Association between maternal dietary fish and cod liver oil whilst breastfeeding and allergic disease / symptoms at 6 years: stratified by sex 14
Table S12: Association between maternal dietary fish and cod liver oil whilst breastfeeding and allergic disease / symptoms at 6 years: subgroup with data collected at 6 weeks 15
Table S13: Association between maternal dietary fish and cod liver oil whilst breastfeeding and allergic disease / symptoms at 6 years: subgroups without symptoms of allergy related disease by 6 month and 1 year of age 16

§3.4. Fish and cod liver oil consumption by infants at 1 year and 2 years and allergy related outcomes 17

1-year analysis (Tables S14 – S16) 17

Table S14: Association between infant dietary fish and cod liver oil at 1 year and allergic disease / symptoms at 6 years: crude and adjusted analyses 17

Table S15: Association between infant dietary fish and cod liver oil at 1 year and allergic disease / symptoms at 6 years: stratified by sex 18

Table S16: Association between infant dietary fish and cod liver oil at 1 year and allergic disease / symptoms at 6 years: subgroups without symptoms of allergy related disease by 6 month and 1 year of age 19

2-year analysis (Tables S17 – S19) 20

Table S17: Association between infant dietary fish and cod liver oil at 2 years and allergic disease / symptoms at 6 years: crude and adjusted analyses 20

Table S18: Association between infant dietary fish and cod liver oil at 2 years and allergic disease / symptoms at 6 years: stratified by sex 21

Table S19: Association between infant dietary fish and cod liver oil at 2 years and allergic disease / symptoms at 6 years: subgroups without symptoms of allergy related disease by 6 month and 1 year of age 22

§2.1. Study Population: Further details of the PACT study recruitment

This is an ancillary study of the PACT study [23]. The PACT study is a controlled, population-based, primary intervention which aimed to reduce the incidence of allergy related diseases through a multifaceted lifestyle intervention during pregnancy and the first 2 years of life. All pregnant women attending routine antenatal care appointments and children attending health checks at 6-weeks, 1 year, 2 years and 6 years were invited to the PACT study.

The control cohort: Participants were included into the control cohort from 1st September 2000. Initially, all pregnant women, and children at ages 6 weeks, 1 year, 2 years and 6 years were invited at routine follow-up with their local GP, midwife or child health centre to complete questionnaires as participants in the control cohort. This made up the comparison population for the intervention cohort, and these were followed until 6 years of age.

The intervention cohort: The intervention was implemented as community health advice to all pregnant mothers in Trondheim from 1st June 2002. Pregnant women who were included after that date belonged to the intervention cohort. Additionally, any child born after 1st January 2003 was considered to belong to the intervention cohort in an "intention to treat" manner, even if the mother had not been included during pregnancy. After the introduction of the intervention, 6-week-old children continued to be recruited to the control cohort until February 2003 since their families would not have received the PACT advice at their initial antenatal visit. Similarly, 1-year-olds were recruited to the control cohort until January 2004 and 2-year-olds until January 2005. Finally, the inclusion of 6-year-olds to the control cohort concluded 30th December 2009 and to the intervention cohort on 12th January 2014. From 2007, the 6-year-old child health questionnaires were distributed through the primary schools rather than through the child health centres.

§3.1. Baseline characteristics of complete cases and drop-outs for each timepoint (Table S1-4)

Table S1: Baseline characteristics for participants included in pregnancy analyses and drop-outs

	**Crude analyses**						**Adjusted analyses (model 1)**					
	Complete cases (n = XXXX)			Drop-outs			Complete cases			Drop-outs		
Covariates	N	n	%	N	n	%	N	n	%	N	n	%
Sex, male	3364	1700	50.5	4061	2037	50.2	2954	1499	50.7	4471	2238	50.1
Family history of allergy	3213	2258	70.3	4546	3338	73.4	2955	2084	70.5	4804	3512	73.1
Older siblings	3218	1818	56.5	4536	2328	51.3	2955	1681	56.9	4799	2465	51.4
Breastfeeding at 6 mo.	2824	2406	85.2	3192	2541	79.6	2560	2193	85.7	3456	2754	79.7
Mother smoking												
Pregnancy	1896	130	6.9	2488	213	8.6	1646	97	5.9	2738	246	9.0
6 weeks	2177	165	7.6	2406	240	10.0	2037	142	7.0	2546	263	10.3
1 year	2312	346	15.0	2685	482	18.0	2155	287	13.3	2842	541	19.0
Pregnancy or first year	3326	464	14.0	4801	754	15.7	2955	372	12.6	5172	846	16.4
Father smoking												
Pregnancy	1852	296	16.0	2395	481	20.1	1628	245	15.0	2619	532	20.3
6 weeks	2071	340	16.4	2300	451	19.6	1984	325	16.4	2387	466	19.5
1 year	2159	370	17.1	2495	507	20.3	2079	351	16.9	2575	526	20.4
Pregnancy or first year	3224	664	20.6	4579	1060	23.1	2955	592	20.0	4848	1132	23.3
Either parent smoking												
Pregnancy	1830	316	17.3	2371	511	21.6	1615	270	16.7	2586	557	21.5
6 weeks	2039	367	18.0	2226	458	20.6	1958	353	18.0	2307	472	20.5
1 year	2139	458	21.4	2464	614	24.9	2069	439	21.2	2534	633	25.0
Pregnancy or first year	3203	777	24.3	4522	1206	26.7	2955	711	24.1	4770	1272	26.7
Cohort, intervention	3367	1195	35.5	4961	1706	34.4	2955	1027	34.8	5373	1874	34.9
	N	mean	SD	N	mean	SD	N	mean	SD	N	mean	SD
Maternal age, yrs	3356	30.3	4.4	3990	29.3	4.7	2955	30.5	4.3	4391	29.3	4.7
Birthweight, gm	3076	3586.4	553.3	3875	3583.0	591.2	2783	3588.1	555.8	4168	3582.0	587.0
Breastfeeding, months	2526	10.6	5.5	2736	9.4	5.7	2286	10.7	5.5	2976	9.4	5.7
Income, NOK	3273	257051	28604	4416	250328	31782	2955	257655	28297	4734	250402	31715
Maternal education, yrs	2360	15.7	2.6	1159	15.5	2.6	2065	15.8	2.6	1454	15.4	2.6
Paternal education, yrs	2332	15.1	2.9	1140	15.1	3.0	2049	15.2	2.9	1423	15.0	3.0

Table S2: Baseline characteristics for participants included in breastfeeding analyses and drop-outs

	Crude analyses						Adjusted analyses (model 1)					
	Complete cases (n = XXXX)			Drop-outs			Complete cases (n = 2955)			Drop-outs		
Covariates	N	n	%	N	n	%	N	n	%	N	n	%
Sex, male	2940	1479	50.3	3851	1966	51.1	2704	1364	50.4	4087	2081	50.9
Family history of allergy	2921	1977	67.7	3836	2617	68.2	2704	1840	68.0	4053	2754	67.9
Older siblings	2928	1660	56.7	3842	2008	52.3	2704	1543	57.1	4066	2125	52.3
Breastfeeding at 6 mo.	2678	2308	86.2	3127	2460	78.7	2471	2138	86.5	3334	2630	78.9
Maternal smoking												
Pregnancy	1506	89	5.9	1440	96	6.7	1410	79	5.6	1536	106	6.9
6 weeks	2155	162	7.5	2428	243	10.0	2017	141	7.0	2566	264	10.3
1 year	2281	335	14.7	2716	493	18.2	2130	279	13.1	2867	549	19.1
Pregnancy or first year	2915	412	14.1	3774	648	17.2	2704	346	12.8	3985	714	17.9
Paternal smoking												
Pregnancy	1473	229	15.5	1391	267	19.2	1393	211	15.1	1471	285	19.4
6 weeks	2052	335	16.3	2319	456	19.7	1967	321	16.3	2404	470	19.6
1 year	2134	362	17.0	2520	515	20.4	2055	344	16.7	2599	533	20.5
Pregnancy or first year	2827	587	20.8	3593	856	23.8	2704	550	20.3	3716	893	24.0
Either parent smoking												
Pregnancy	1458	246	16.9	1379	276	20.0	1380	228	16.5	1457	294	20.2
6 weeks	2022	363	18.0	2243	462	20.6	1941	349	18.0	2324	476	20.5
1 year	2115	449	21.2	2488	623	25.0	2045	430	21.0	2558	642	25.1
Pregnancy or first year	2815	697	24.8	3546	981	27.7	2704	659	24.4	3657	1019	27.9
Cohort, intervention	2940	939	31.9	3858	998	25.9	2704	874	32.3	4094	1063	26.0
	N	mean	SD	N	mean	SD	N	mean	SD	N	mean	SD
Maternal age, yrs	2935	30.4	4.4	3839	29.3	4.7	2704	30.5	4.3	4070	29.2	4.7
Birthweight, gm	2925	3585.6	554.3	3815	3578.1	594.1	2692	3588.5	554.5	4048	3576.6	591.7
Breastfeeding, months	2384	10.7	5.4	2671	9.2	5.7	2198	10.8	5.4	2857	9.2	5.7
Income, NOK	2860	257103	28305	3527	250661	31773	2704	257614	28061	3683	250558	31742
Maternal education, yrs	1982	15.7	2.6	956	15.6	2.6	1846	15.8	2.6	1092	15.4	2.6
Paternal education, yrs	1959	15.1	2.9	943	15.1	3.0	1831	15.2	2.9	1071	14.9	3.0

Table S3: Baseline characteristics for participants included in 1-year analyses and drop-outs

	Crude analyses						Adjusted analyses (model 1)					
	Complete cases (n = XXXX)			Drop-outs			Complete cases (n = 2955)			Drop-outs		
Covariates	N	n	%	N	n	%	N	n	%	N	n	%
Sex, male	2352	1169	49.7	2761	1396	50.6	2176	1087	50.0	2937	1478	50.3
Family history of allergy	2340	1586	67.8	2751	1858	67.5	2176	1479	68.0	2915	1965	67.4
Older siblings	2347	1328	56.6	2754	1464	53.2	2176	1237	56.8	2925	1555	53.2
Breastfeeding at 6 mo.	2347	1993	84.9	2736	2224	81.3	2173	1857	85.5	2910	2360	81.1
Maternal smoking												
Pregnancy	1123	70	6.2	878	50	5.7	1057	61	5.8	944	59	6.3
6 weeks	1565	115	7.3	1428	124	8.7	1482	101	6.8	1511	138	9.1
1 year	2318	348	15.0	2679	480	17.9	2159	289	13.4	2838	539	19.0
Pregnancy or first year	2338	369	15.8	2720	503	18.5	2176	307	14.1	2882	565	19.6
Paternal smoking												
Pregnancy	1096	179	16.3	853	152	17.8	1047	168	16.0	902	163	18.1
6 weeks	1493	235	15.7	1373	253	18.4	1441	228	15.8	1425	260	18.2
1 year	2163	371	17.2	2491	506	20.3	2083	352	16.9	2571	525	20.4
Pregnancy or first year	2261	479	21.2	2590	607	23.4	2176	452	20.8	2675	634	23.7
Either parent smoking												
Pregnancy	1085	193	17.8	845	159	18.8	1036	181	17.5	894	171	19.1
6 weeks	1468	256	17.4	1336	259	19.4	1419	249	17.5	1385	266	19.2
1 year	2143	460	21.5	2460	612	24.9	2073	441	21.3	2530	631	24.9
Pregnancy or first year	2251	580	25.8	2570	722	28.1	2176	552	25.4	2645	750	28.4
Cohort, intervention	2352	649	27.6	2763	591	21.4	2176	606	27.8	2939	634	21.6
	N	mean	SD	N	mean	SD	N	mean	SD	N	mean	SD
Maternal age, yrs	2347	30.3	4.4	2749	29.4	4.6	2176	30.4	4.3	2920	29.3	4.7
Birthweight, gm	2339	3574.6	560.8	2731	3575.2	600.3	2166	3579.2	563.2	2904	3571.8	596.4
Breastfeeding, months	2061	10.4	5.4	2289	9.4	5.5	1908	10.5	5.4	2442	9.4	5.5
Income, NOK	2294	256211	28402	2552	250390	31563	2176	256804	27972	2670	250164	31672
Maternal education, yrs	1524	15.7	2.6	682	15.6	2.7	1428	15.8	2.6	778	15.4	2.7
Paternal education, yrs	1505	15.1	3.0	673	15.1	3.0	1416	15.2	3.0	762	14.9	3.1

Table S4: Baseline characteristics for participants included in 2-year analyses and drop-outs

	Crude analyses						Adjusted analyses (model 1)					
	Complete cases (n = XXXX)			Drop-outs			Complete cases (n = 2955)			Drop-outs		
Covariates	N	n	%	N	n	%	N	n	%	N	n	%
Sex, male	3039	1519	50.0	3166	1572	49.7	1952	1010	51.7	4253	2081	48.9
Family history of allergy	2104	1446	68.7	1835	1312	71.5	1952	1345	68.9	1987	1413	71.1
Older siblings	2109	1175	55.7	1840	1005	54.6	1952	1097	56.2	1997	1083	54.2
Breastfeeding at 6 mo.	3013	2582	85.7	3128	2589	82.8	1947	1695	87.1	4194	3476	82.9
Maternal smoking												
Pregnancy	1271	73	5.7	844	56	6.6	1145	60	5.2	970	69	7.1
6 weeks	1530	101	6.6	1115	95	8.5	1432	90	6.3	1213	106	8.7
1 year	1657	225	13.6	1459	247	16.9	1558	189	12.1	1558	283	18.2
Pregnancy or first year	2129	274	12.9	1845	293	15.9	1952	229	11.7	2022	338	16.7
Paternal smoking												
Pregnancy	1248	180	14.4	819	136	16.6	1133	155	13.7	934	161	17.2
6 weeks	1473	216	14.7	1065	194	18.2	1397	203	14.5	1141	207	18.1
1 year	1564	244	15.6	1367	275	20.1	1506	230	15.3	1425	289	20.3
Pregnancy or first year	2089	394	18.9	1769	396	22.4	1952	354	18.1	1906	436	22.9
Either parent smoking												
Pregnancy	1234	190	15.4	810	138	17.0	1124	168	14.9	920	160	17.4
6 weeks	1448	234	16.2	1039	192	18.5	1378	222	16.1	1109	204	18.4
1 year	1550	302	19.5	1347	323	24.0	1497	288	19.2	1400	337	24.1
Pregnancy or first year	2077	467	22.5	1750	449	25.7	1952	431	22.1	1875	485	25.9
Cohort, intervention	3039	819	26.9	3168	579	18.3	1952	733	37.6	4255	665	15.6
	N	mean	SD	N	mean	SD	N	mean	SD	N	mean	SD
Maternal age, yrs	3030	30.3	4.4	3148	29.5	4.7	1952	30.5	4.3	4226	29.7	4.6
Birthweight, gm	3007	3586.8	560.3	3107	3586.5	600.6	1948	3591.0	548.5	4166	3584.7	595.8
Breastfeeding, months	2943	11.2	5.6	3064	10.6	5.6	1894	11.4	5.6	4113	10.7	5.6
Income, NOK	2949	256588	27690	2983	251808	30638	1952	256532	27297	3980	253032	30177
Maternal education, yrs	1768	15.7	2.5	880	15.6	2.6	1410	15.8	2.5	1238	15.5	2.6
Paternal education, yrs	1752	15.1	2.9	878	15.1	2.9	1402	15.1	2.8	1228	15.1	3.0

§3.2. Maternal and Infant Dietary Fish and Cod Liver Oil Intake: Description and Correlations

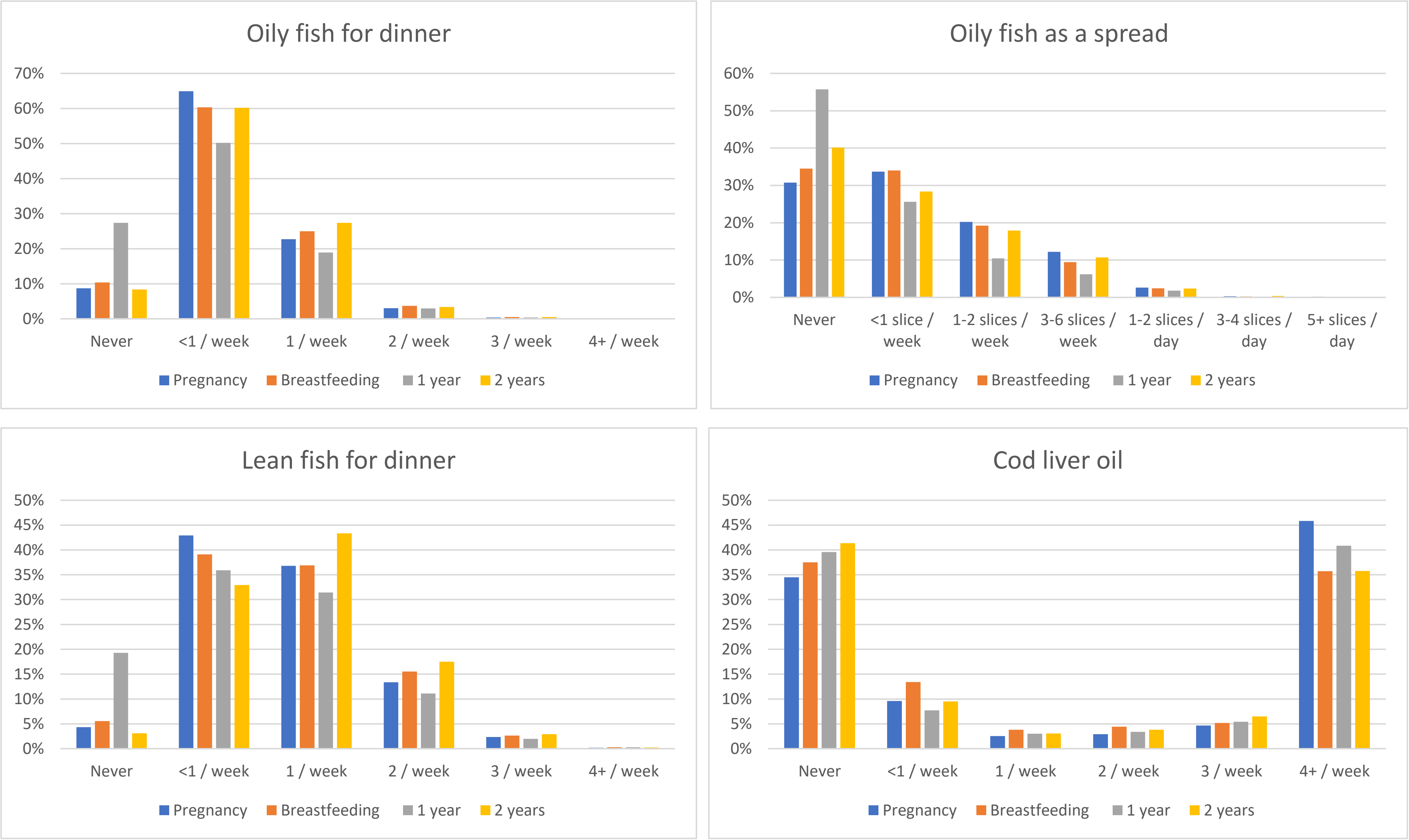

Figure S1: Distribution of fish and cod liver oil consumption

Table S5: Correlation / agreement between diet at different time points

	N	Kendall's tau-b	ASE	Agreement	RR*	95% CI		
**Any fish**								
Pregnancy - Breastfeeding	6255	0.659	0.010	84.3	3.83	3.55	-	4.13
Pregnancy - 1 year	4911	0.254	0.014	62.6	1.76	1.64	-	1.89
Pregnancy - 2 years	3888	0.322	0.016	69.5	1.55	1.48	-	1.64
Breastfeeding - 1 year	4738	0.263	0.014	62.9	1.82	1.69	-	1.96
Breastfeeding - 2 years	3533	0.315	0.017	70.0	1.55	1.46	-	1.63
1 year - 2 years	3102	0.275	0.017	62.8	1.41	1.35	-	1.48
**Fat fish**								
Pregnancy - Breastfeeding	6289	0.637	0.010	83.1	5.48	5.07	-	5.93
Pregnancy - 1 year	4924	0.291	0.014	69.1	2.53	2.31	-	2.78
Pregnancy - 2 years	3897	0.295	0.016	66.2	1.97	1.83	-	2.11
Breastfeeding - 1 year	4759	0.308	0.014	69.4	2.69	2.45	-	2.95
Breastfeeding - 2 years	3550	0.313	0.016	66.9	2.05	1.90	-	2.21
1 year - 2 years	3108	0.326	0.017	68.0	2.12	1.97	-	2.29
**Lean fish**								
Pregnancy - Breastfeeding	6612	0.668	0.009	83.5	4.36	4.06	-	4.69
Pregnancy - 1 year	5041	0.284	0.013	63.7	1.96	1.82	-	2.10
Pregnancy - 2 years	3971	0.360	0.015	68.1	1.78	1.69	-	1.88
Breastfeeding - 1 year	4954	0.301	0.013	64.3	2.07	1.92	-	2.23
Breastfeeding - 2 years	3702	0.343	0.016	67.8	1.74	1.65	-	1.85
1 year - 2 years	3125	0.288	0.017	62.6	1.55	1.47	-	1.64
**Cod liver oil**								
Pregnancy - Breastfeeding	6591	0.637	0.008	72.9				
Pregnancy - 1 year	5049	0.265	0.013	52.0				
Pregnancy - 2 years	3971	0.259	0.014	50.1				
Breastfeeding - 1 year	4950	0.300	0.012	52.8				
Breastfeeding - 2 years	3704	0.298	0.014	51.0				
1 year - 2 years	3132	0.494	0.014	62.2				

ASE: Asymptotic standard error; RR: relative risk; These relative risk indicate the relationship between fish consumption at the earlier timepoint as a predictor of fish consumption at the later timepoint (i.e. women who ate fish during pregnancy were 3.83 times more likely to eat fish whilst breastfeeding).

Table S6: The proportion of families consuming fish at differing combinations of pregnancy, breastfeeding and 1 year

	Any fish (n=2202)		Oily fish (n=2211)		Lean fish (n=2295)	
	n	%	n	%	n	%
None	404	18.35	1,040	47.04	608	26.49
Preg only	97	4.41	131	5.92	108	4.71
Preg & BF	459	20.84	300	13.57	417	18.17
Preg & 1yr	68	3.09	56	2.53	54	2.35
All	798	36.24	293	13.25	665	28.98
BF only	88	4.00	132	5.97	116	5.05
BF & 1yr	87	3.95	79	3.57	100	4.36
1yr only	201	9.13	180	8.14	227	9.89

BF: breastfeeding

§3.3. Maternal fish and cod liver oil intake whilst pregnant allergy related outcomes at 6 years (Table S7-9)

Table S7: Association between maternal dietary fish and cod liver oil in pregnancy and allergic disease / symptoms at 6 years: crude and adjusted analyses

	Crude OR				Adjusted Model 1				Adjusted Model 2			
	N	OR	95% CI	p-value	N	aOR	95% CI	p-value	N	aOR	95% CI	p-value
**Any fish, >= 1 vs < 1 time per week**												
Current eczema	3245	0.92	0.75-1.13	0.426	2858	0.91	0.73-1.14	0.407	2012	0.95	0.67-1.35	0.789
Current asthma	3299	1.07	0.75-1.52	0.706	2901	1.20	0.82-1.75	0.358	2046	1.83	1.01-3.32	0.047
Current wheeze	3265	0.97	0.77-1.23	0.807	2870	1.02	0.79-1.32	0.869	2024	1.15	0.76-1.74	0.517
ARC	3066	1.15	0.92-1.45	0.221	2696	1.24	0.97-1.60	0.088	1916	1.36	0.91-2.02	0.133
**Oily fish, >= 1 vs < 1 time per week**												
Current eczema	3248	0.89	0.72-1.10	0.287	2861	0.89	0.71-1.12	0.307	2018	0.97	0.69-1.36	0.844
Current asthma	3302	1.14	0.81-1.60	0.449	2904	1.34	0.94-1.92	0.111	2053	1.66	0.96-2.89	0.071
Current wheeze	3268	1.14	0.91-1.44	0.252	2873	1.27	1.00-1.63	0.054	2031	1.32	0.90-1.93	0.155
ARC	3069	1.16	0.93-1.44	0.188	2699	1.23	0.97-1.56	0.087	1922	1.62	1.13-2.33	0.009
**Lean fish, >= 1 vs < 1 time per week**												
Current eczema	3306	1.03	0.84-1.26	0.768	2907	1.00	0.80-1.24	0.968	2093	0.98	0.70-1.39	0.931
Current asthma	3360	1.07	0.77-1.48	0.691	2950	1.16	0.81-1.66	0.411	2128	1.61	0.91-2.85	0.104
Current wheeze	3326	0.96	0.77-1.20	0.696	2919	0.97	0.76-1.24	0.824	2105	1.05	0.71-1.55	0.826
ARC	3123	1.04	0.84-1.29	0.708	2741	1.07	0.84-1.35	0.583	1994	0.97	0.67-1.40	0.851
**Cod liver oil**												
Current eczema, 1-3 / week vs never	3307	1.06	0.79-1.42	0.693	2907	1.08	0.79-1.47	0.629	2057	1.10	0.74-1.64	0.633
Current eczema, 4+ / week vs never	3307	1.22	0.97-1.52	0.086	2907	1.23	0.95-1.57	0.112	2057	1.25	0.85-1.84	0.255
Current asthma, 1-3 / week vs never	3361	0.56	0.33-0.94	0.029	2950	0.57	0.32-1.01	0.053	2093	0.44	0.20-0.95	0.036
Current asthma, 4+ / week vs never	3361	0.81	0.58-1.15	0.249	2950	0.96	0.65-1.42	0.828	2093	0.86	0.47-1.60	0.643
Current wheeze, 1-3 / week vs never	3327	1.01	0.74-1.39	0.933	2919	0.99	0.70-1.39	0.936	2070	1.08	0.70-1.67	0.716
Current wheeze, 4+ / week vs never	3327	0.92	0.72-1.18	0.519	2919	1.05	0.80-1.38	0.715	2070	1.18	0.76-1.82	0.459
ARC, 1-3 / week vs never	3124	0.99	0.73-1.33	0.924	2741	1.10	0.80-1.53	0.561	1960	1.17	0.77-1.78	0.452
ARC, 4+ / week vs never	3124	0.88	0.70-1.12	0.304	2741	1.01	0.77-1.33	0.917	1960	1.17	0.78-1.77	0.450

ARC: allergic rhinoconjunctivitis; Model 1 adjusted for family history of atopy, presence of older siblings, parental smoking within during pregnancy and the first year of life, maternal age, average income of postal code area and interventional cohort. Model 2 for any fish, oily fish and lean fish models: adjusted as for model 1 + cod liver oil consumption in pregnancy and any fish during breastfeeding (mother) and at 1 year (infant); Model 2 and for cod liver oil models: adjusted as for model 1 + any fish consumption in pregnancy and cod liver oil during breastfeeding (mother) and at 1 year (infant).

Table S8: Association between maternal dietary fish and cod liver oil in pregnancy and allergic disease / symptoms at 6 years: stratified by sex

	Boys				Girls				Total			
	N	aOR	95% CI	p-value	N	aOR	95% CI	p-value	N	aOR	95% CI	p-value
**Any fish, >= 1 vs < 1 time per week**												
Current eczema	1442	0.78	0.56-1.09	0.153	1415	1.04	0.77-1.42	0.788	2858	0.91	0.73-1.14	0.407
Current asthma	1468	1.19	0.73-1.94	0.484	1432	1.12	0.61-2.07	0.719	2901	1.20	0.82-1.75	0.358
Current wheeze	1447	1.05	0.75-1.48	0.763	1422	0.95	0.65-1.40	0.796	2870	1.02	0.79-1.32	0.869
ARC	1359	1.29	0.92-1.79	0.137	1337	1.12	0.76-1.66	0.560	2696	1.24	0.97-1.60	0.088
**Fatty fish, >= 1 vs < 1 time per week**												
Current eczema	1444	0.82	0.58-1.15	0.247	1416	0.94	0.69-1.28	0.696	2861	0.89	0.71-1.12	0.307
Current asthma	1470	1.24	0.79-1.94	0.359	1433	1.46	0.80-2.65	0.218	2904	1.34	0.94-1.92	0.111
Current wheeze	1449	1.22	0.88-1.68	0.231	1423	1.32	0.90-1.94	0.156	2873	1.27	1.00-1.63	0.054
ARC	1361	1.20	0.88-1.63	0.250	1338	1.27	0.86-1.85	0.227	2699	1.23	0.97-1.56	0.087
**Lean fish, >= 1 vs < 1 time per week**												
Current eczema	1469	0.99	0.72-1.37	0.969	1437	1.01	0.75-1.36	0.932	2907	1.00	0.80-1.24	0.968
Current asthma	1495	1.34	0.85-2.11	0.213	1454	0.88	0.49-1.58	0.658	2950	1.16	0.81-1.66	0.411
Current wheeze	1474	1.02	0.74-1.40	0.910	1444	0.89	0.62-1.29	0.549	2919	0.97	0.76-1.24	0.824
ARC	1384	1.10	0.81-1.49	0.556	1357	0.97	0.67-1.41	0.878	2741	1.07	0.84-1.35	0.583
**Cod liver oil**												
Current eczema, 1-3 / week vs never	1469	0.97	0.61-1.55	0.915	1437	1.16	0.76-1.76	0.497	2907	1.08	0.79-1.47	0.629
Current eczema, 4+ / week vs never	1469	0.97	0.67-1.40	0.882	1437	1.52	1.07-2.15	0.019	2907	1.23	0.95-1.57	0.112
Current asthma, 1-3 / week vs never	1495	0.48	0.23-1.02	0.056	1454	0.78	0.32-1.91	0.580	2950	0.57	0.32-1.01	0.053
Current asthma, 4+ / week vs never	1495	0.81	0.50-1.32	0.398	1454	1.30	0.67-2.54	0.438	2950	0.96	0.65-1.42	0.828
Current wheeze, 1-3 / week vs never	1474	0.94	0.60-1.48	0.801	1444	1.07	0.63-1.83	0.792	2919	0.99	0.70-1.39	0.936
Current wheeze, 4+ / week vs never	1474	0.82	0.57-1.18	0.282	1444	1.50	0.98-2.30	0.065	2919	1.05	0.80-1.38	0.715
ARC, 1-3 / week vs never	1384	1.09	0.71-1.69	0.685	1357	1.17	0.71-1.92	0.534	2741	1.10	0.80-1.53	0.561
ARC, 4+ / week vs never	1384	1.08	0.76-1.52	0.670	1357	0.92	0.59-1.42	0.692	2741	1.01	0.77-1.33	0.917

ARC: allergic rhinoconjunctivitis; All models adjusted for family history of atopy, presence of older siblings, parental smoking within during pregnancy and the first year of life, maternal age, average income of postal code area and interventional cohort (Model 1).

Table S9: Association between maternal dietary fish and cod liver oil in pregnancy and allergic disease / symptoms at 6 years: subgroup with data collected during pregnancy

	Pregnancy questionnaire only				Total			
	N	aOR	95% CI	p-value	N	aOR	95% CI	p-value
**Any fish, >= 1 vs < 1 time per week**								
Current eczema	1689	0.81	0.61-1.07	0.142	2858	0.91	0.73-1.14	0.407
Current asthma	1713	1.22	0.74-2.03	0.435	2901	1.20	0.82-1.75	0.358
Current wheeze	1698	0.99	0.71-1.38	0.948	2870	1.02	0.79-1.32	0.869
ARC	1586	1.17	0.85-1.63	0.334	2696	1.24	0.97-1.60	0.088
**Fatty fish, >= 1 vs < 1 time per week**								
Current eczema	1689	0.81	0.60-1.08	0.149	2861	0.89	0.71-1.12	0.307
Current asthma	1714	1.11	0.68-1.82	0.670	2904	1.34	0.94-1.92	0.111
Current wheeze	1699	1.04	0.75-1.46	0.803	2873	1.27	1.00-1.63	0.054
ARC	1587	1.07	0.78-1.48	0.667	2699	1.23	0.97-1.56	0.087
**Lean fish, >= 1 vs < 1 time per week**								
Current eczema	1696	0.88	0.67-1.16	0.378	2907	1.00	0.8-1.24	0.968
Current asthma	1720	1.31	0.81-2.11	0.276	2950	1.16	0.81-1.66	0.411
Current wheeze	1705	1.10	0.8-1.53	0.554	2919	0.97	0.76-1.24	0.824
ARC	1592	1.00	0.73-1.37	0.991	2741	1.07	0.84-1.35	0.583
**Cod liver oil**								
Current eczema, 1-3 / week vs never	1689	0.99	0.66-1.47	0.947	2907	1.08	0.79-1.47	0.629
Current eczema, 4+ / week vs never	1689	1.19	0.86-1.65	0.295	2907	1.23	0.95-1.57	0.112
Current asthma, 1-3 / week vs never	1714	0.58	0.27-1.22	0.150	2950	0.57	0.32-1.01	0.053
Current asthma, 4+ / week vs never	1714	0.85	0.50-1.46	0.557	2950	0.96	0.65-1.42	0.828
Current wheeze, 1-3 / week vs never	1699	1.10	0.70-1.73	0.695	2919	0.99	0.70-1.39	0.936
Current wheeze, 4+ / week vs never	1699	1.08	0.73-1.59	0.695	2919	1.05	0.80-1.38	0.715
ARC, 1-3 / week vs never	1586	0.86	0.56-1.32	0.493	2741	1.10	0.80-1.53	0.561
ARC, 4+ / week vs never	1586	0.74	0.51-1.06	0.101	2741	1.01	0.77-1.33	0.917

ARC: allergic rhinoconjunctivitis; All models adjusted for family history of atopy, presence of older siblings, parental smoking within during pregnancy and the first year of life, maternal age, average income of postal code area and interventional cohort (Model 1).

§3.4. Maternal fish and cod liver oil intake whilst breastfeeding and allergy related outcomes at 6 years (Tables 10-13)

Table S10: Association between maternal dietary fish and cod liver oil whilst breastfeeding and allergic disease / symptoms at 6 years: crude and adjusted analyses

	Crude				Adjusted (Model 1)				Adjusted (Model 2)			
	N	aOR	95% CI	p-value	N	aOR	95% CI	p-value	N	aOR	95% CI	p-value
**Any fish, >= 1 vs < 1 time per week**												
Current eczema	2739	0.92	0.74-1.16	0.502	2529	0.94	0.73-1.20	0.592	2006	1.00	0.70-1.43	0.982
Current asthma	2780	0.89	0.61-1.29	0.526	2567	0.91	0.61-1.35	0.627	2040	0.69	0.39-1.22	0.202
Current wheeze	2752	1.04	0.80-1.34	0.789	2540	1.08	0.82-1.42	0.608	2018	1.18	0.78-1.79	0.433
ARC	2590	0.87	0.68-1.11	0.261	2391	0.94	0.72-1.23	0.657	1910	0.89	0.60-1.31	0.555
**Fatty fish, >= 1 vs < 1 time per week**												
Current eczema	2751	0.80	0.64-1.01	0.063	2539	0.79	0.62-1.01	0.059	2011	0.76	0.53-1.08	0.121
Current asthma	2793	1.12	0.78-1.61	0.539	2578	1.09	0.74-1.61	0.670	2046	0.94	0.54-1.66	0.841
Current wheeze	2765	1.14	0.89-1.46	0.304	2551	1.23	0.94-1.6	0.125	2024	1.24	0.84-1.82	0.280
ARC	2602	0.96	0.75-1.22	0.733	2401	0.94	0.73-1.23	0.671	1915	0.72	0.49-1.05	0.086
**Lean fish, >= 1 vs < 1 time per week**												
Current eczema	2876	1.05	0.85-1.31	0.644	2647	1.05	0.83-1.32	0.697	2083	1.13	0.80-1.59	0.493
Current asthma	2919	0.93	0.66-1.32	0.690	2686	1.01	0.70-1.47	0.943	2118	0.83	0.47-1.48	0.536
Current wheeze	2890	0.98	0.77-1.25	0.894	2658	1.03	0.80-1.33	0.823	2095	1.21	0.81-1.81	0.342
ARC	2720	0.93	0.74-1.17	0.536	2502	1.05	0.82-1.35	0.680	1984	1.30	0.89-1.90	0.167
**Cod liver oil**												
Current eczema, 1-3 / week vs never	2870	0.96	0.71-1.3	0.794	2645	0.89	0.65-1.23	0.490	2014	0.80	0.53-1.22	0.305
Current eczema, 4+ / week vs never	2870	1.19	0.93-1.52	0.162	2645	1.20	0.92-1.57	0.186	2014	1.15	0.76-1.73	0.505
Current asthma, 1-3 / week vs never	2913	0.87	0.55-1.39	0.563	2685	0.89	0.54-1.46	0.646	2048	0.94	0.49-1.81	0.862
Current asthma, 4+ / week vs never	2913	0.84	0.57-1.24	0.376	2685	0.90	0.58-1.40	0.644	2048	1.06	0.54-2.05	0.872
Current wheeze, 1-3 / week vs never	2884	1.04	0.76-1.42	0.823	2657	1.10	0.79-1.53	0.579	2026	1.03	0.66-1.59	0.901
Current wheeze, 4+ / week vs never	2884	0.87	0.66-1.14	0.301	2657	0.99	0.73-1.33	0.935	2026	1.03	0.65-1.64	0.884
ARC, 1-3 / week vs never	2716	0.92	0.68-1.25	0.609	2502	1.02	0.74-1.42	0.895	1918	0.86	0.56-1.32	0.500
ARC, 4+ / week vs never	2716	0.89	0.69-1.16	0.394	2502	0.97	0.73-1.30	0.844	1918	0.85	0.55-1.33	0.481

ARC: allergic rhinoconjunctivitis; Model 1 adjusted for family history of atopy, presence of older siblings, parental smoking within during pregnancy and the first year of life, maternal age, average income of postal code area and interventional cohort. Model 2 for any fish, oily fish and lean fish models: adjusted as for model 1 + cod liver oil consumption whilst breastfeeding and any fish during pregnancy (mother) and at 1 year (infant); Model 2 and for cod liver oil models: adjusted as for model 1 + any fish consumption whilst breastfeeding and cod liver oil during pregnancy (mother) and at 1 year (infant).

Table S11: Association between maternal dietary fish and cod liver oil whilst breastfeeding and allergic disease / symptoms at 6 years: stratified by sex

	Boys				Girls				Total			
	N	aOR	95% CI	p-value	N	aOR	95% CI	p-value	N	aOR	95% CI	p-value
**Any fish, >= 1 vs < 1 time per week**												
Current eczema	1264	0.92	0.63-1.32	0.644	1265	0.97	0.69-1.35	0.846	2529	0.94	0.73-1.20	0.592
Current asthma	1286	0.81	0.49-1.34	0.407	1281	1.02	0.53-1.96	0.951	2567	0.91	0.61-1.35	0.627
Current wheeze	1268	1.05	0.73-1.52	0.779	1272	1.08	0.71-1.65	0.724	2540	1.08	0.82-1.42	0.608
ARC	1193	0.89	0.63-1.26	0.521	1198	0.97	0.64-1.48	0.887	2391	0.94	0.72-1.23	0.657
**Fatty fish, >= 1 vs < 1 time per week**												
Current eczema	1272	0.78	0.54-1.13	0.192	1267	0.79	0.57-1.11	0.173	2539	0.79	0.62-1.01	0.059
Current asthma	1295	0.86	0.52-1.42	0.547	1283	1.53	0.82-2.86	0.183	2578	1.09	0.74-1.61	0.670
Current wheeze	1277	1.16	0.82-1.65	0.397	1274	1.32	0.87-1.99	0.192	2551	1.23	0.94-1.60	0.125
ARC	1201	0.89	0.63-1.25	0.493	1200	1.03	0.68-1.56	0.902	2401	0.94	0.73-1.23	0.671
**Lean fish, >= 1 vs < 1 time per week**												
Current eczema	1328	1.03	0.73-1.45	0.881	1319	1.08	0.79-1.48	0.624	2647	1.05	0.83-1.32	0.697
Current asthma	1351	1.09	0.68-1.76	0.721	1335	0.85	0.46-1.58	0.615	2686	1.01	0.70-1.47	0.943
Current wheeze	1332	1.02	0.73-1.43	0.909	1326	1.04	0.70-1.54	0.835	2658	1.03	0.80-1.33	0.823
ARC	1254	1.03	0.75-1.42	0.865	1248	1.05	0.71-1.56	0.803	2502	1.05	0.82-1.35	0.680
**Cod liver oil**												
Current eczema, 1-3 / week vs never	1332	0.89	0.55-1.43	0.630	1313	0.90	0.58-1.40	0.637	2645	0.89	0.65-1.23	0.490
Current eczema, 4+ / week vs never	1332	0.91	0.61-1.37	0.656	1313	1.55	1.07-2.23	0.019	2645	1.20	0.92-1.57	0.186
Current asthma, 1-3 / week vs never	1356	0.60	0.30-1.19	0.142	1329	1.51	0.71-3.22	0.283	2685	0.89	0.54-1.46	0.646
Current asthma, 4+ / week vs never	1356	0.85	0.50-1.45	0.552	1329	0.96	0.44-2.06	0.907	2685	0.90	0.58-1.40	0.644
Current wheeze, 1-3 / week vs never	1337	0.80	0.51-1.26	0.333	1320	1.65	1.00-2.72	0.051	2657	1.10	0.79-1.53	0.579
Current wheeze, 4+ / week vs never	1337	0.75	0.51-1.12	0.163	1320	1.39	0.87-2.23	0.170	2657	0.99	0.73-1.33	0.935
ARC, 1-3 / week vs never	1259	0.99	0.64-1.52	0.966	1243	1.05	0.63-1.75	0.841	2502	1.02	0.74-1.42	0.895
ARC, 4+ / week vs never	1259	0.99	0.68-1.45	0.977	1243	0.89	0.56-1.42	0.622	2502	0.97	0.73-1.30	0.844

ARC: allergic rhinoconjunctivitis; All models adjusted for family history of atopy, presence of older siblings, parental smoking within during pregnancy and the first year of life, maternal age, average income of postal code area and interventional cohort (Model 1).

Table S12: Association between maternal dietary fish and cod liver oil whilst breastfeeding and allergic disease / symptoms at 6 years: subgroup with data collected at 6 weeks

	6-week questionnaire only				Total			
	N	aOR	95% CI	p-value	N	aOR	95% CI	p-value
**Any fish, >= 1 vs < 1 time per week**								
Current eczema	1694	0.97	0.72-1.32	0.867	2529	0.94	0.73-1.20	0.592
Current asthma	1715	0.82	0.51-1.31	0.402	2567	0.91	0.61-1.35	0.627
Current wheeze	1701	1.04	0.74-1.46	0.810	2540	1.08	0.82-1.42	0.608
ARC	1586	0.73	0.53-1.00	0.050	2391	0.94	0.72-1.23	0.657
**Fatty fish, >= 1 vs < 1 time per week**								
Current eczema	1705	0.73	0.54-0.99	0.041	2539	0.79	0.62-1.01	0.059
Current asthma	1726	1.07	0.67-1.72	0.778	2578	1.09	0.74-1.61	0.670
Current wheeze	1712	1.21	0.88-1.67	0.244	2551	1.23	0.94-1.60	0.125
ARC	1596	0.85	0.61-1.17	0.317	2401	0.94	0.73-1.23	0.671
**Lean fish, >= 1 vs < 1 time per week**								
Current eczema	1842	1.05	0.80-1.38	0.708	2647	1.05	0.83-1.32	0.697
Current asthma	1865	0.85	0.54-1.32	0.460	2686	1.01	0.70-1.47	0.943
Current wheeze	1850	0.91	0.67-1.23	0.532	2658	1.03	0.80-1.33	0.823
ARC	1722	0.79	0.59-1.07	0.125	2502	1.05	0.82-1.35	0.680
**Cod liver oil**								
Current eczema, 1-3 / week vs never	1843	0.81	0.55-1.20	0.301	2645	0.89	0.65-1.23	0.490
Current eczema, 4+ / week vs never	1843	1.12	0.82-1.55	0.472	2645	1.20	0.92-1.57	0.186
Current asthma, 1-3 / week vs never	1866	1.01	0.55-1.85	0.966	2685	0.89	0.54-1.46	0.646
Current asthma, 4+ / week vs never	1866	1.01	0.60-1.71	0.973	2685	0.90	0.58-1.40	0.644
Current wheeze, 1-3 / week vs never	1851	1.24	0.83-1.87	0.293	2657	1.10	0.79-1.53	0.579
Current wheeze, 4+ / week vs never	1851	1.08	0.75-1.56	0.662	2657	0.99	0.73-1.33	0.935
ARC, 1-3 / week vs never	1723	1.01	0.68-1.49	0.979	2502	1.02	0.74-1.42	0.895
ARC, 4+ / week vs never	1723	0.85	0.60-1.21	0.359	2502	0.97	0.73-1.30	0.844

RC: allergic rhinoconjunctivitis; All models adjusted for family history of atopy, presence of older siblings, parental smoking within during pregnancy and the first year of life, maternal age, average income of postal code area and interventional cohort (Model 1).

Table S13: Association between maternal dietary fish and cod liver oil whilst breastfeeding and allergic disease / symptoms at 6 years: subgroups without symptoms of allergy related disease by 6 month and 1 year of age

	Symptoms before 6 mo. excluded				Symptoms before 1yr excluded				Total			
	N	aOR	95% CI	p-value	N	aOR	95% CI	p-value	N	aOR	95% CI	p-value
**Any fish, >= 1 vs < 1 time per week**												
Current eczema	2192	0.99	0.73-1.35	0.952	1900	1.18	0.79-1.75	0.417	2529	0.94	0.73-1.20	0.592
Current asthma	2219	0.95	0.59-1.51	0.821	1919	1.21	0.62-2.34	0.576	2567	0.91	0.61-1.35	0.627
Current wheeze	2196	1.26	0.92-1.74	0.155	1901	1.25	0.86-1.82	0.251	2540	1.08	0.82-1.42	0.608
ARC	2071	1.26	0.91-1.76	0.166	1802	1.28	0.87-1.87	0.211	2391	0.94	0.72-1.23	0.657
**Fatty fish, >= 1 vs < 1 time per week**												
Current eczema	2200	0.86	0.64-1.17	0.345	1906	0.74	0.50-1.10	0.134	2539	0.79	0.62-1.01	0.059
Current asthma	2228	1.05	0.67-1.67	0.823	1925	0.80	0.43-1.50	0.490	2578	1.09	0.74-1.61	0.670
Current wheeze	2205	1.34	1.00-1.80	0.052	1907	1.51	1.07-2.13	0.019	2551	1.23	0.94-1.60	0.125
ARC	2079	1.26	0.93-1.72	0.135	1808	1.29	0.91-1.83	0.158	2401	0.94	0.73-1.23	0.671
**Lean fish, >= 1 vs < 1 time per week**												
Current eczema	2294	1.12	0.84-1.49	0.429	1991	1.41	0.98-2.05	0.066	2647	1.05	0.83-1.32	0.697
Current asthma	2322	1.12	0.72-1.75	0.618	2011	1.47	0.80-2.72	0.218	2686	1.01	0.70-1.47	0.943
Current wheeze	2298	1.19	0.89-1.60	0.234	1992	1.11	0.79-1.55	0.563	2658	1.03	0.80-1.33	0.823
ARC	2168	1.33	0.98-1.80	0.063	1888	1.30	0.92-1.83	0.137	2502	1.05	0.82-1.35	0.680
**Cod liver oil**												
Current eczema, 1-3 / week vs never	2292	0.87	0.58-1.3	0.495	1988	0.65	0.39-1.10	0.110	2645	0.89	0.65-1.23	0.490
Current eczema, 4+ / week vs never	2292	1.24	0.89-1.72	0.203	1988	1.08	0.72-1.62	0.705	2645	1.20	0.92-1.57	0.186
Current asthma, 1-3 / week vs never	2321	0.70	0.38-1.28	0.249	2008	0.83	0.38-1.83	0.648	2685	0.89	0.54-1.46	0.646
Current asthma, 4+ / week vs never	2321	0.81	0.49-1.34	0.413	2008	0.86	0.44-1.69	0.659	2685	0.90	0.58-1.40	0.644
Current wheeze, 1-3 / week vs never	2297	1.07	0.74-1.55	0.728	1989	0.84	0.54-1.32	0.451	2657	1.10	0.79-1.53	0.579
Current wheeze, 4+ / week vs never	2297	1.01	0.72-1.41	0.949	1989	0.87	0.59-1.28	0.471	2657	0.99	0.73-1.33	0.935
ARC, 1-3 / week vs never	2168	0.97	0.67-1.43	0.893	1887	1.10	0.72-1.68	0.670	2502	1.02	0.74-1.42	0.895
ARC, 4+ / week vs never	2168	0.79	0.56-1.12	0.191	1887	0.80	0.54-1.19	0.272	2502	0.97	0.73-1.30	0.844

ARC: allergic rhinoconjunctivitis; All models adjusted for family history of atopy, presence of older siblings, parental smoking within during pregnancy and the first year of life, maternal age, average income of postal code area and interventional cohort (Model 1).

§3.4. Fish and cod liver oil consumption by infants at 1 year and 2 years and allergy related outcomes

1-year analysis (Tables S14 – S16)

Table S14: Association between infant dietary fish and cod liver oil at 1 year and allergic disease / symptoms at 6 years: crude and adjusted analyses

	Crude				Adjusted (Model 1)				Adjusted (Model 2)			
	N	aOR	95% CI	p-value	N	aOR	95% CI	p-value	N	aOR	95% CI	p-value
**Any fish, >= 1 vs < 1 time per week**												
Current eczema	2293	0.73	0.57-0.93	0.011	2125	0.72	0.56-0.93	0.011	2008	0.69	0.53-0.91	0.009
Current asthma	2335	0.58	0.39-0.87	0.008	2162	0.60	0.39-0.91	0.018	2041	0.55	0.35-0.87	0.011
Current wheeze	2312	0.61	0.47-0.80	0.000	2139	0.66	0.50-0.88	0.005	2019	0.62	0.45-0.83	0.002
ARC	2185	0.93	0.72-1.20	0.568	2023	0.92	0.70-1.21	0.534	1911	0.86	0.64-1.16	0.322
**Fatty fish, >= 1 vs < 1 time per week**												
Current eczema	2294	1.03	0.78-1.34	0.857	2126	0.95	0.71-1.26	0.713	2014	1.01	0.74-1.38	0.949
Current asthma	2336	0.71	0.44-1.14	0.159	2163	0.72	0.43-1.20	0.211	2048	0.66	0.38-1.15	0.144
Current wheeze	2313	0.74	0.54-1.02	0.069	2140	0.83	0.60-1.16	0.276	2026	0.74	0.51-1.06	0.099
ARC	2186	1.11	0.84-1.46	0.465	2024	1.07	0.79-1.45	0.657	1917	1.05	0.75-1.46	0.781
**Lean fish, >= 1 vs < 1 time per week**												
Current eczema	2297	0.74	0.58-0.94	0.015	2129	0.76	0.59-0.99	0.043	2090	0.74	0.57-0.98	0.036
Current asthma	2339	0.54	0.36-0.83	0.004	2166	0.56	0.36-0.88	0.012	2124	0.55	0.34-0.88	0.013
Current wheeze	2316	0.65	0.49-0.85	0.002	2143	0.69	0.52-0.92	0.012	2101	0.66	0.48-0.89	0.007
ARC	2189	0.86	0.66-1.10	0.230	2027	0.85	0.65-1.12	0.250	1990	0.79	0.59-1.06	0.117
**Cod liver oil**												
Current eczema, 1-3 / week vs never	2300	0.81	0.59-1.13	0.220	2131	0.85	0.60-1.20	0.352	2077	0.81	0.57-1.15	0.229
Current eczema, 4+ / week vs never	2300	0.71	0.54-0.93	0.014	2131	0.71	0.53-0.95	0.022	2077	0.64	0.47-0.87	0.005
Current asthma, 1-3 / week vs never	2342	1.09	0.66-1.81	0.736	2168	1.13	0.66-1.92	0.657	2112	1.21	0.70-2.10	0.486
Current asthma, 4+ / week vs never	2342	0.76	0.48-1.19	0.226	2168	0.69	0.42-1.13	0.138	2112	0.74	0.44-1.26	0.268
Current wheeze, 1-3 / week vs never	2319	0.90	0.63-1.29	0.568	2145	0.95	0.65-1.38	0.776	2089	0.99	0.67-1.45	0.941
Current wheeze, 4+ / week vs never	2319	0.77	0.57-1.04	0.088	2145	0.78	0.57-1.08	0.142	2089	0.80	0.57-1.14	0.218
ARC, 1-3 / week vs never	2191	0.96	0.68-1.36	0.814	2029	1.00	0.69-1.46	0.981	1977	1.02	0.70-1.49	0.920
ARC, 4+ / week vs never	2191	1.03	0.78-1.37	0.827	2029	1.04	0.76-1.42	0.790	1977	1.05	0.75-1.47	0.772

ARC: allergic rhinoconjunctivitis; Model 1 adjusted for family history of atopy, presence of older siblings, parental smoking within during pregnancy and the first year of life, maternal age, average income of postal code area and interventional cohort. Model 2 for any fish, oily fish and lean fish models: adjusted as for model 1 + breastfeeding at 6 months, cod liver oil consumption at 2 years and any fish consumption during pregnancy and breastfeeding (mother); Model 2 and for cod liver oil models: adjusted as for model 1 + breastfeeding at 6 months, any fish consumption at 2 years and cod liver oil during pregnancy and breastfeeding (mother).

Table S15: Association between infant dietary fish and cod liver oil at 1 year and allergic disease / symptoms at 6 years: stratified by sex

	Boys				Girls				Total			
	N	aOR	95% CI	p-value	N	aOR	95% CI	p-value	N	aOR	95% CI	p-value
**Any fish, >= 1 vs < 1 time per week**												
Current eczema	1056	0.73	0.49-1.07	0.109	1069	0.74	0.52-1.04	0.083	2125	0.72	0.56-0.93	0.011
Current asthma	1078	0.55	0.32-0.95	0.032	1084	0.66	0.33-1.32	0.237	2162	0.60	0.39-0.91	0.018
Current wheeze	1062	0.55	0.38-0.81	0.002	1077	0.82	0.53-1.27	0.375	2139	0.66	0.50-0.88	0.005
ARC	1010	0.79	0.55-1.12	0.189	1013	1.10	0.71-1.71	0.662	2023	0.92	0.70-1.21	0.534
**Fatty fish, >= 1 vs < 1 time per week**												
Current eczema	1057	0.96	0.62-1.47	0.836	1069	0.95	0.64-1.41	0.803	2126	0.95	0.71-1.26	0.713
Current asthma	1079	0.62	0.32-1.20	0.153	1084	0.84	0.37-1.90	0.680	2163	0.72	0.43-1.20	0.211
Current wheeze	1063	0.78	0.51-1.21	0.269	1077	0.87	0.51-1.46	0.595	2140	0.83	0.60-1.16	0.276
ARC	1011	0.86	0.58-1.29	0.467	1013	1.40	0.88-2.24	0.160	2024	1.07	0.79-1.45	0.657
**Lean fish, >= 1 vs < 1 time per week**												
Current eczema	1059	0.82	0.56-1.22	0.325	1070	0.75	0.53-1.06	0.104	2129	0.76	0.59-0.99	0.043
Current asthma	1081	0.57	0.32-0.99	0.045	1085	0.53	0.25-1.11	0.092	2166	0.56	0.36-0.88	0.012
Current wheeze	1065	0.57	0.39-0.84	0.005	1078	0.86	0.55-1.33	0.486	2143	0.69	0.52-0.92	0.012
ARC	1013	0.79	0.55-1.13	0.191	1014	0.88	0.57-1.37	0.579	2027	0.85	0.65-1.12	0.250
**Cod liver oil**												
Current eczema, 1-3 / week vs never	1057	1.01	0.59-1.72	0.979	1074	0.77	0.49-1.2	0.249	2131	0.85	0.60-1.20	0.352
Current eczema, 4+ / week vs never	1057	0.89	0.57-1.39	0.602	1074	0.62	0.42-0.91	0.015	2131	0.71	0.53-0.95	0.022
Current asthma, 1-3 / week vs never	1079	1.16	0.58-2.32	0.667	1089	1.06	0.46-2.46	0.888	2168	1.13	0.66-1.92	0.657
Current asthma, 4+ / week vs never	1079	0.74	0.39-1.39	0.351	1089	0.55	0.24-1.28	0.167	2168	0.69	0.42-1.13	0.138
Current wheeze, 1-3 / week vs never	1063	0.85	0.51-1.43	0.551	1082	1.11	0.64-1.93	0.706	2145	0.95	0.65-1.38	0.776
Current wheeze, 4+ / week vs never	1063	0.80	0.52-1.24	0.319	1082	0.74	0.45-1.23	0.246	2145	0.78	0.57-1.08	0.142
ARC, 1-3 / week vs never	1011	0.79	0.47-1.33	0.372	1018	1.31	0.76-2.26	0.339	2029	1.00	0.69-1.46	0.981
ARC, 4+ / week vs never	1011	1.21	0.81-1.80	0.360	1018	0.76	0.45-1.28	0.305	2029	1.04	0.76-1.42	0.790

ARC: allergic rhinoconjunctivitis; All models adjusted for family history of atopy, presence of older siblings, parental smoking within during pregnancy and the first year of life, maternal age, average income of postal code area and interventional cohort (Model 1).

Table S16: Association between infant dietary fish and cod liver oil at 1 year and allergic disease / symptoms at 6 years: subgroups without symptoms of allergy related disease by 6 month and 1 year of age

	Symptoms before 6 mo. excluded				Symptoms before 1yr excluded				Total			
	N	aOR	95% CI	p-value	N	aOR	95% CI	p-value	N	aOR	95% CI	p-value
**Any fish, >= 1 vs < 1 time per week**												
Current eczema	1836	0.72	0.52-0.99	0.043	1584	0.76	0.51-1.14	0.182	2125	0.72	0.56-0.93	0.011
Current asthma	1863	0.60	0.36-0.98	0.041	1604	0.59	0.30-1.17	0.131	2162	0.60	0.39-0.91	0.018
Current wheeze	1843	0.80	0.58-1.09	0.156	1589	0.81	0.56-1.17	0.259	2139	0.66	0.50-0.88	0.005
ARC	1744	1.04	0.75-1.44	0.824	1510	1.31	0.89-1.93	0.169	2023	0.92	0.70-1.21	0.534
**Fatty fish, >= 1 vs < 1 time per week**												
Current eczema	1837	0.90	0.63-1.30	0.586	1585	0.90	0.57-1.43	0.663	2126	0.95	0.71-1.26	0.713
Current asthma	1864	0.74	0.41-1.33	0.317	1605	0.71	0.32-1.58	0.400	2163	0.72	0.43-1.20	0.211
Current wheeze	1844	0.94	0.65-1.35	0.733	1590	1.00	0.66-1.52	0.991	2140	0.83	0.60-1.16	0.276
ARC	1745	1.10	0.77-1.58	0.588	1511	1.29	0.86-1.93	0.221	2024	1.07	0.79-1.45	0.657
**Lean fish, >= 1 vs < 1 time per week**												
Current eczema	1839	0.76	0.55-1.05	0.092	1586	0.75	0.50-1.13	0.168	2129	0.76	0.59-0.99	0.043
Current asthma	1866	0.50	0.30-0.85	0.010	1606	0.43	0.20-0.89	0.024	2166	0.56	0.36-0.88	0.012
Current wheeze	1846	0.82	0.60-1.12	0.207	1591	0.80	0.55-1.15	0.223	2143	0.69	0.52-0.92	0.012
ARC	1747	0.97	0.70-1.35	0.871	1512	1.15	0.79-1.67	0.470	2027	0.85	0.65-1.12	0.250
**Cod liver oil**												
Current eczema, 1-3 / week vs never	1840	1.04	0.69-1.56	0.849	1587	0.83	0.49-1.40	0.476	2131	0.85	0.60-1.20	0.352
Current eczema, 4+ / week vs never	1840	0.71	0.49-1.02	0.067	1587	0.58	0.36-0.93	0.024	2131	0.71	0.53-0.95	0.022
Current asthma, 1-3 / week vs never	1868	1.04	0.56-1.96	0.895	1608	0.89	0.36-2.24	0.807	2168	1.13	0.66-1.92	0.657
Current asthma, 4+ / week vs never	1868	0.69	0.39-1.24	0.216	1608	0.75	0.35-1.61	0.455	2168	0.69	0.42-1.13	0.138
Current wheeze, 1-3 / week vs never	1848	0.86	0.56-1.32	0.493	1593	0.98	0.60-1.60	0.939	2145	0.95	0.65-1.38	0.776
Current wheeze, 4+ / week vs never	1848	0.88	0.61-1.25	0.468	1593	0.89	0.59-1.35	0.586	2145	0.78	0.57-1.08	0.142
ARC, 1-3 / week vs never	1749	1.01	0.65-1.57	0.968	1514	0.85	0.50-1.45	0.558	2029	1.00	0.69-1.46	0.981
ARC, 4+ / week vs never	1749	1.01	0.69-1.47	0.960	1514	1.05	0.69-1.61	0.805	2029	1.04	0.76-1.42	0.790

ARC: allergic rhinoconjunctivitis; All models adjusted for family history of atopy, presence of older siblings, parental smoking within during pregnancy and the first year of life, maternal age, average income of postal code area and interventional cohort (Model 1).

2-year analysis (Tables S17 – S19)

Table S17: Association between infant dietary fish and cod liver oil at 2 years and allergic disease / symptoms at 6 years: crude and adjusted analyses

	Crude				Adjusted (Model 1)				Adjusted (Model 2)			
	N	aOR	95% CI	p-value	N	aOR	95% CI	p-value	N	aOR	95% CI	p-value
**Any fish, >= 1 vs < 1 time per week**												
Current eczema	2963	1.07	0.85-1.35	0.577	1900	1.19	0.87-1.61	0.272	1453	1.28	0.88-1.88	0.201
Current asthma	3011	0.96	0.66-1.4	0.848	1932	1.09	0.66-1.81	0.727	1481	1.07	0.58-1.96	0.836
Current wheeze	2980	1.05	0.81-1.37	0.706	1913	1.31	0.93-1.86	0.123	1465	1.10	0.72-1.67	0.669
ARC	2787	0.88	0.69-1.12	0.299	1793	1.01	0.73-1.39	0.955	1380	0.94	0.63-1.41	0.767
**Fatty fish, >= 1 vs < 1 time per week**												
Current eczema	2967	1.06	0.86-1.31	0.601	1903	1.13	0.86-1.48	0.374	1458	1.16	0.82-1.63	0.401
Current asthma	3015	0.94	0.67-1.34	0.744	1935	1.08	0.69-1.69	0.752	1487	1.25	0.71-2.19	0.433
Current wheeze	2984	1.22	0.96-1.54	0.101	1916	1.37	1.01-1.84	0.041	1471	1.19	0.82-1.74	0.363
ARC	2791	0.82	0.65-1.04	0.099	1796	0.90	0.67-1.20	0.466	1385	0.76	0.53-1.11	0.159
**Lean fish, >= 1 vs < 1 time per week**												
Current eczema	2973	0.98	0.79-1.22	0.876	1907	1.05	0.8-1.39	0.704	1495	1.09	0.77-1.54	0.622
Current asthma	3022	0.95	0.67-1.35	0.761	1939	1.07	0.68-1.70	0.766	1524	1.00	0.57-1.75	0.996
Current wheeze	2990	1.07	0.84-1.37	0.587	1919	1.41	1.03-1.95	0.033	1507	1.25	0.85-1.83	0.265
ARC	2796	0.93	0.74-1.18	0.560	1799	1.04	0.78-1.39	0.792	1421	1.03	0.72-1.48	0.871
**Cod liver oil**												
Current eczema, 1-3 / week vs never	2968	1.10	0.84-1.44	0.500	1908	1.07	0.76-1.51	0.695	1495	1.17	0.77-1.77	0.456
Current eczema, 4+ / week vs never	2968	1.00	0.79-1.27	0.990	1908	0.95	0.69-1.30	0.732	1495	0.90	0.59-1.37	0.623
Current asthma, 1-3 / week vs never	3017	0.82	0.51-1.30	0.391	1940	1.03	0.58-1.82	0.925	1526	1.22	0.63-2.36	0.559
Current asthma, 4+ / week vs never	3017	0.97	0.66-1.42	0.859	1940	0.86	0.51-1.46	0.574	1526	0.84	0.42-1.68	0.622
Current wheeze, 1-3 / week vs never	2986	0.84	0.61-1.16	0.290	1921	0.85	0.57-1.27	0.423	1509	0.87	0.54-1.39	0.562
Current wheeze, 4+ / week vs never	2986	1.02	0.78-1.32	0.908	1921	0.98	0.69-1.38	0.888	1509	0.93	0.59-1.46	0.757
ARC, 1-3 / week vs never	2792	1.17	0.87-1.57	0.302	1801	1.09	0.76-1.57	0.645	1422	1.00	0.65-1.55	0.988
ARC, 4+ / week vs never	2792	1.12	0.86-1.45	0.390	1801	0.97	0.69-1.36	0.861	1422	0.78	0.50-1.22	0.273

ARC: allergic rhinoconjunctivitis; Model 1 adjusted for family history of atopy, presence of older siblings, parental smoking within during pregnancy and the first year of life, maternal age, average income of postal code area and interventional cohort. Model 2 for any fish, oily fish and lean fish models: adjusted as for model 1 + breastfeeding at 6 months, cod liver oil consumption at 2 years and any fish consumption during pregnancy and breastfeeding (mother); Model 2 and for cod liver oil models: adjusted as for model 1 + breastfeeding at 6 months, any fish consumption at 2 years and cod liver oil during pregnancy and breastfeeding (mother).

Table S18: Association between infant dietary fish and cod liver oil at 2 years and allergic disease / symptoms at 6 years: stratified by sex

	Boys				Girls				Total			
	N	aOR	95% CI	p-value	N	aOR	95% CI	p-value	N	aOR	95% CI	p-value
**Any fish, >= 1 vs < 1 time per week**												
Current eczema	980	0.97	0.63-1.5	0.902	920	1.42	0.92-2.18	0.109	1900	1.19	0.87-1.61	0.272
Current asthma	1001	0.77	0.43-1.36	0.364	931	3.03	0.9-10.23	0.074	1932	1.09	0.66-1.81	0.727
Current wheeze	988	1.04	0.67-1.60	0.873	925	1.96	1.07-3.59	0.030	1913	1.31	0.93-1.86	0.123
ARC	925	0.81	0.54-1.22	0.312	868	1.43	0.85-2.43	0.180	1793	1.01	0.73-1.39	0.955
**Fatty fish, >= 1 vs < 1 time per week**												
Current eczema	982	0.94	0.63-1.40	0.762	921	1.32	0.91-1.91	0.139	1903	1.13	0.86-1.48	0.374
Current asthma	1003	0.77	0.44-1.37	0.377	932	2.02	0.91-4.48	0.082	1935	1.08	0.69-1.69	0.752
Current wheeze	990	1.02	0.69-1.51	0.905	926	2.10	1.30-3.39	0.003	1916	1.37	1.01-1.84	0.041
ARC	927	0.73	0.49-1.07	0.108	869	1.21	0.77-1.90	0.398	1796	0.90	0.67-1.2	0.466
**Lean fish, >= 1 vs < 1 time per week**												
Current eczema	983	0.98	0.66-1.47	0.935	924	1.13	0.78-1.65	0.511	1907	1.05	0.8-1.39	0.704
Current asthma	1004	0.82	0.47-1.43	0.489	935	1.80	0.75-4.32	0.191	1939	1.07	0.68-1.70	0.766
Current wheeze	991	1.20	0.80-1.80	0.383	928	1.79	1.06-3.02	0.031	1919	1.41	1.03-1.95	0.033
ARC	928	0.85	0.58-1.24	0.404	871	1.36	0.85-2.17	0.195	1799	1.04	0.78-1.39	0.792
**Cod liver oil**												
Current eczema, 1-3 / week vs never	982	0.76	0.46-1.27	0.302	926	1.44	0.90-2.30	0.131	1908	1.07	0.76-1.51	0.695
Current eczema, 4+ / week vs never	982	0.70	0.44-1.12	0.137	926	1.23	0.80-1.90	0.341	1908	0.95	0.69-1.3	0.732
Current asthma, 1-3 / week vs never	1003	0.61	0.27-1.35	0.221	937	1.90	0.78-4.63	0.155	1940	1.03	0.58-1.82	0.925
Current asthma, 4+ / week vs never	1003	1.04	0.56-1.93	0.907	937	0.46	0.15-1.40	0.173	1940	0.86	0.51-1.46	0.574
Current wheeze, 1-3 / week vs never	990	0.76	0.46-1.28	0.303	931	1.00	0.53-1.89	0.988	1921	0.85	0.57-1.27	0.423
Current wheeze, 4+ / week vs never	990	0.88	0.56-1.39	0.587	931	1.12	0.65-1.94	0.673	1921	0.98	0.69-1.38	0.888
ARC, 1-3 / week vs never	927	0.78	0.48-1.28	0.330	874	1.66	0.95-2.91	0.077	1801	1.09	0.76-1.57	0.645
ARC, 4+ / week vs never	927	0.87	0.57-1.35	0.540	874	1.12	0.65-1.92	0.695	1801	0.97	0.69-1.36	0.861

ARC: allergic rhinoconjunctivitis; All models adjusted for family history of atopy, presence of older siblings, parental smoking within during pregnancy and the first year of life, maternal age, average income of postal code area and interventional cohort (Model 1).

Table S19: Association between infant dietary fish and cod liver oil at 2 years and allergic disease / symptoms at 6 years: subgroups without symptoms of allergy related disease by 6 month and 1 year of age

	Symptoms before 6 mo. excluded				Symptoms before 1yr excluded				Total			
	N	aOR	95% CI	p-value	N	aOR	95% CI	p-value	N	aOR	95% CI	p-value
**Any fish, >= 1 vs < 1 time per week**												
Current eczema	1611	1.19	0.81-1.74	0.375	1376	1.17	0.72-1.90	0.516	1900	1.19	0.87-1.61	0.272
Current asthma	1634	1.40	0.73-2.69	0.311	1394	2.21	0.84-5.78	0.107	1932	1.09	0.66-1.81	0.727
Current wheeze	1617	1.84	1.20-2.82	0.005	1382	2.09	1.23-3.53	0.006	1913	1.31	0.93-1.86	0.123
ARC	1523	1.07	0.73-1.58	0.720	1308	1.17	0.74-1.85	0.501	1793	1.01	0.73-1.39	0.955
**Fatty fish, >= 1 vs < 1 time per week**												
Current eczema	1613	1.13	0.81-1.58	0.469	1377	1.25	0.82-1.92	0.297	1903	1.13	0.86-1.48	0.374
Current asthma	1636	1.07	0.62-1.86	0.803	1395	0.89	0.44-1.80	0.747	1935	1.08	0.69-1.69	0.752
Current wheeze	1619	1.61	1.14-2.26	0.006	1383	1.82	1.22-2.73	0.004	1916	1.37	1.01-1.84	0.041
ARC	1525	0.96	0.68-1.37	0.839	1309	1.11	0.74-1.65	0.628	1796	0.90	0.67-1.20	0.466
**Lean fish, >= 1 vs < 1 time per week**												
Current eczema	1617	1.00	0.72-1.41	0.978	1381	0.89	0.58-1.36	0.596	1907	1.05	0.8-1.39	0.704
Current asthma	1640	1.14	0.64-2.00	0.659	1399	1.43	0.68-3.03	0.348	1939	1.07	0.68-1.70	0.766
Current wheeze	1622	1.69	1.16-2.46	0.006	1386	1.70	1.09-2.65	0.019	1919	1.41	1.03-1.95	0.033
ARC	1528	1.07	0.75-1.53	0.694	1312	1.11	0.73-1.67	0.634	1799	1.04	0.78-1.39	0.792
**Cod liver oil**												
Current eczema, 1-3 / week vs never	1618	1.01	0.66-1.54	0.957	1381	0.74	0.42-1.30	0.294	1908	1.07	0.76-1.51	0.695
Current eczema, 4+ / week vs never	1618	0.87	0.59-1.29	0.496	1381	0.99	0.61-1.60	0.965	1908	0.95	0.69-1.30	0.732
Current asthma, 1-3 / week vs never	1641	0.89	0.45-1.75	0.729	1399	0.80	0.33-1.95	0.629	1940	1.03	0.58-1.82	0.925
Current asthma, 4+ / week vs never	1641	0.56	0.29-1.10	0.094	1399	0.59	0.26-1.37	0.220	1940	0.86	0.51-1.46	0.574
Current wheeze, 1-3 / week vs never	1624	0.85	0.54-1.33	0.477	1387	0.69	0.4-1.18	0.172	1921	0.85	0.57-1.27	0.423
Current wheeze, 4+ / week vs never	1624	0.98	0.67-1.46	0.937	1387	0.86	0.54-1.36	0.512	1921	0.98	0.69-1.38	0.888
ARC, 1-3 / week vs never	1530	1.08	0.69-1.67	0.746	1313	1.03	0.62-1.70	0.923	1801	1.09	0.76-1.57	0.645
ARC, 4+ / week vs never	1530	0.98	0.65-1.46	0.905	1313	0.92	0.58-1.48	0.741	1801	0.97	0.69-1.36	0.861

ARC: allergic rhinoconjunctivitis; All models adjusted for family history of atopy, presence of older siblings, parental smoking within during pregnancy and the first year of life, maternal age, average income of postal code area and interventional cohort (Model 1).
